# TBSV Alters Host Redox State After Short-Term Temperature Pre-Exposure in *Nicotiana benthamiana*

**DOI:** 10.3390/biom16030446

**Published:** 2026-03-17

**Authors:** Ulbike Amanbayeva, Assemgul Bekturova, Assylay Kurmanbayeva, Tetiana Todosiichuk, Almas Madirov, Zhibek Turarbekova, Mereke Satkanov, Zhaksylyk Masalimov

**Affiliations:** 1 Department of Biotechnology and Microbiology, L.N. Gumilyov Eurasian National University, Astana 010000, Kazakhstan; 2 Laboratory of Molecular Genetics and Genomics, Zhetysu University named after I. Zhansugurov, Taldykorgan 040009, Kazakhstan; 3 Laboratory of Biodiversity and Genetic Resources, National Center for Biotechnology, Astana 010000, Kazakhstan; 4 Faculty of Biotechnology and Biotechnics, National Technical University of Ukraine “Igor Sikorsky Kyiv Polytechnic Institute”, 03056 Kyiv, Ukraine; 5 LLP “Biosense Research Institute”, Ust-Kamenogorsk 070000, Kazakhstan

**Keywords:** abiotic stress pre-exposure, redox homeostasis, viral pathogenesis, plant–virus interactions, redox-related enzyme response

## Abstract

Plant viruses can cause substantial yield losses, yet disease severity often varies between seasons because plants frequently experience heat or cold episodes before infection. In this study, we tested whether such temperature conditions affect the plant’s redox balance and alter its response to *Tomato bushy stunt virus* (TBSV) infection in *Nicotiana benthamiana*. Plants were exposed to short-term heat and cold stress, after which they recovered before virus inoculation. Following this, we assessed the reactive oxygen species (ROS) content, lipid peroxidation (LPO), oxidative DNA damage, stress-related proteins, redox-associated enzymes, and antioxidant metabolites. TBSV led to non-parallel ROS responses during infection, with consistently elevated hydrogen peroxide in infected plants but reduced superoxide relative to corresponding mock controls. Heat pre-exposure caused pronounced LPO that decreased further after infection, whereas cold pre-exposure stabilized malondialdehyde near levels observed at 25 °C. Both thermal stress and infection increased 8-oxo-dG and were associated with distinct changes in 8-oxoguanine glycosylase abundance. Infection strongly induced heat shock protein 90 (and moderately heat shock protein 70), while prior heat limited further chaperone induction by TBSV. These results indicate that viral infection develops within and is limited by the host’s oxidative state, where redox homeostasis may restrict infection-related processes, and infection leads to changes in this redox environment that are favorable for its development.

## 1. Introduction

Plant viruses cause substantial agricultural losses by reducing yield and product quality, largely due to their rapid evolution, efficient vector transmission, and limited control options [1,2,3]. Mechanistically, viruses are obligate intracellular pathogens that can replicate and spread only by exploiting host metabolic resources and regulatory pathways [4,5]. Thus, viral infection induces coordinated changes in host signaling pathways, metabolism, and immunity for its own development [4,5]. Therefore, understanding which host processes promote resistance or, conversely, enable effective infection of plants is crucial. This is of fundamental importance both for plant pathology and for the development of resilient production systems.

A crucial aspect that connects environmental perception with defense and metabolic adaptation is the regulation of redox balance [6]. Viral infection frequently disrupts reactive oxygen species (ROS) homeostasis, and these changes can have two closely related consequences [7,8,9]. On the one hand, ROS are involved in antiviral signaling and can contribute to the activation of defense mechanisms [7,8,9]. On the other hand, excessive or poorly controlled ROS accumulation has been shown to promote oxidative damage to membranes, proteins, and nucleic acids, thereby increasing the metabolic burden of infection and potentially influencing symptom development [7,8,9]. Accordingly, antiviral responses are closely associated with antioxidant systems, including enzymatic components and non-enzymatic metabolites [10,11,12]. Importantly, the outcome of an infection is determined not only by changes in ROS levels but also by the regulatory mechanisms that maintain redox balance and the effectiveness of defense systems in mitigating oxidative damage while preserving signaling functions [10,11,12].

These redox-dependent interactions occur under environmental conditions that vary significantly between real ecosystems and controlled lab settings [13]. Temperature is a key factor influencing plant physiology and redox homeostasis, thereby shaping the development of viral diseases [14]. Short-term periods of heat or cold have been demonstrated to alter ROS production, antioxidant capacity, membrane stability, and stress-response protein systems [10,15]. Consequently, the same infection may originate from different redox baselines in the field, resulting in variable symptom development and yield impacts across seasons and locations [5,16]. Climate variability and extreme temperature events have been observed to intensify combined abiotic and biotic stresses, leading to unpredictable disease outcomes and unstable crop yields, which makes this topic particularly relevant for sustainable agriculture [17,18,19]. Thus, a mechanistic understanding of temperature-dependent virus–host interactions has the potential to inform the development of strategies aimed at maintaining productivity under climate change [20,21,22].

Despite substantial progress in understanding plant–virus interactions, particularly the dual role of ROS as both secondary messengers for antiviral signaling and byproducts of metabolic disturbance, the use of ROS-related responses as a generic indicator of oxidative stress remains a subject of frequent discussion [7]. Specifically, this progress is evident in the discovery of ROS-mediated regulation of RNA silencing in *tobacco mosaic virus* models and in the identification of virus-induced pexophagy that leads to redox imbalance and ferroptosis through peroxisomal catalase degradation [7,8]. This complexity arises because individual ROS species differ in biochemical origin, subcellular localization, mobility, and biological function, meaning their accumulation does not always correlate linearly with the severity of infection [23,24,25]. At the same time, the extent to which prior thermal exposure affects the plant redox state and modifies defense responses during subsequent viral challenge remains insufficiently understood. As a result, it remains unclear whether heat or cold pre-exposure before infection increases oxidative damage or instead forms a distinct physiological context during infection.

In the present study, we addressed this gap by examining how short-term heat and cold pre-exposure influence redox regulation and stress responses during subsequent viral infection. We selected *Nicotiana benthamiana* as a model host plant, given its well-studied redox plasticity and its wide use in phytovirology [26,27]. *Tomato bushy stunt virus* (TBSV) was chosen as a representative RNA virus due to its ability to interact with host redox and chaperone systems without causing rapid tissue damage [28,29,30]. We integrated multiple indicators of redox state, oxidative damage, and protective responses to assess how temperature pretreatment modifies host physiology during infection. Our results indicate that prior thermal exposure is associated with altered ROS regulation, damage-related markers, and stress-protective responses. Collectively, these data provide a comprehensive understanding of the influence of environmental factors on the course of virus-induced responses.

## 2. Materials and Methods

### 2.1. N. benthamiana Growth Conditions

*N. benthamiana* seeds were obtained from the L.N. Gumilyov Eurasian National University (Astana, Kazakhstan) collection. Aseptic seeds were sown in 200 mL plastic pots filled with a sterile soil substrate (a mixture of peat and vermiculite, 3:1 *v*/*v*; pH 6.0–6.5). To ensure uniform development, only one plant was grown per pot. The plants were grown for 30 days with a 16 h light and 8 h dark photoperiod, a light intensity of 150 µmol m^−2^ s^−1^, and relative humidity maintained at 60%, at a temperature of 25 ± 2 °C. Then, temperature treatment was carried out at +40 °C (heat stress) [31] or +10 °C (cold stress) [32] for 24 h, while control plants were kept at +25 °C (optimal temperature) [33]. These temperatures were chosen as contrasting non-lethal cold and heat stress conditions for *N. benthamiana*, sufficient to induce various physiological stress responses while allowing recovery prior to inoculation. All plants were subsequently returned to +25 °C for a 24 h recovery period before inoculation. TBSV- or mock-inoculation was then performed, and plants were further grown at +25 °C. The final assessment was performed for 7 days after inoculation, when leaf samples were collected for biochemical and enzymatic analyses.

### 2.2. Plants Infection with TBSV

Infection with the TBSV virus was carried out according to the generally accepted standardized method with minor modifications [34]. Infectious RNA transcripts of TBSV were synthesized in vitro from full-length cDNA clones (pTBSV100) after plasmid linearization at the 3′ end with SmaI. The transcription reaction was carried out using T7 RNA polymerase according to established protocols. For plant infection, 1 μg of the resulting transcripts was diluted in phosphate buffer (10 mM, pH 6.9–7.0) at a 1:3 ratio. Thirty-day-old *N. benthamiana* plants were then inoculated by manually rubbing the mixture into the third tier of leaves. To facilitate virus penetration, 0.001% carborundum (Sigma-Aldrich, Vienna, Austria) was used as an abrasive during the procedure. Control plants not inoculated with the virus were similarly treated with phosphate buffer and carborundum in the absence of viral transcripts.

### 2.3. Histochemical Staining for ROS Assessment

Histochemical NBT and DAB staining were used for superoxide (O_2_^•−^) and hydrogen peroxide (H_2_O_2_) visualization [35]. For quantification, images were converted to grayscale, inverted, and analyzed by gray value quantification in ImageJ [36]. Mock-inoculated plants grown at +25 °C were used as the control, and relative staining intensity was expressed as a percentage of the control (100%).

### 2.4. DNA and Membranes Oxidative Damage Assessment

Oxidative damage to DNA and membranes was assessed by quantifying 8-oxoguanine (8-oxo-dG) and malondialdehyde (MDA) contents. Total genomic DNA was isolated from leaves using a CTAB-based protocol, and 8-oxo-dG levels were determined via ELISA and measured at 450 nm [37]. Lipid peroxidation (LPO) was assessed using the thiobarbituric acid assay, with absorbance measured at 532 and 600 nm, and MDA equivalents were expressed on a fresh-weight basis [38].

### 2.5. Western Blot Analysis

Assessment of 8-oxoguanine glycosylase (OGG1) activity and abundance of heat shock proteins (HSPs) was performed via Western blot analysis. OGG1 was detected using anti-OGG1 polyclonal antibodies (Cusabio, Houston, TX, USA) according to the manufacturer’s recommendations [39]. For HSP analysis, immunoblotting was performed as previously described [27]. In both cases, total leaf proteins were separated by SDS-PAGE and transferred to membranes using a semi-dry blotting system. Membranes were blocked and incubated with primary antibodies, diluted 1:10,000, followed by incubation with secondary antibodies diluted 1:5000. Protein transfer and immunoblot analysis were performed twice, and reproducible banding patterns were obtained in independent experiments. Immunoreactive bands were visualized. Band intensities were quantified using ImageJ 1.54g [40] and GelAnalyzer 23.1. [41] software and expressed relative to the control (%).

### 2.6. Redox-Associated Enzyme Activities Assessment

Aldehyde oxidase (AO) [42], superoxide dismutase (SOD) [43], catalase (CAT) [44], and peroxidase (POD) [45] were detected using native-PAGE in-gel activities assays. Total soluble proteins were extracted from fresh leaf, quantified via the Bradford assay using bovine serum albumin as a standard, and equal protein amounts were separated on 7.5% native polyacrylamide gels [42,46]. Gels were stained using substrate-specific procedures, documented under white light, and relative band intensities were quantified using ImageJ [40] and GelAnalyzer [41] software and expressed relative to the control (%).

Glutathione reductase (GR) activity was assessed with a commercial assay kit (Abcam) according to the manufacturer’s instructions [47]. The assay is based on the GR-dependent reduction of oxidized glutathione (GSSG) to GSH, followed by reaction with DTNB and measurement of absorbance at 405 nm. GR activity was expressed as mU/mL.

### 2.7. Determination of the Content of Phenolic Compounds

Total phenolic content (TPC) was determined using the Folin–Ciocalteu method, with absorbance measured at 765 nm and results expressed as gallic acid equivalents [48]. Total flavonoids were quantified with the aluminum chloride colorimetric assay, measured at 400 nm, and results were calculated as cynaroside equivalents [49]. Anthocyanin content was determined after extraction in an acidified solution, with absorbance measured at 530 and 657 nm and calculated according to a standard equation [50].

### 2.8. Non-Enzymatic Antioxidant Content Assessment

The ascorbic acid (AsA) content was determined spectrophotometrically using the method of Hewitt and Dickes (1961) [51]. Fresh leaf tissue was extracted with metaphosphoric acid, and the absorbance of the clarified extract was measured at 265 nm. The AsA concentration was calculated using the molar extinction coefficient and expressed on a per-fresh-weight basis. The free proline content was determined using the ninhydrin method, as described by Bates et al. (1973) with slight modifications [38,52]. Absorbance was measured at 520 nm, and proline concentration was calculated from a standard curve and expressed per gram of fresh weight. α-tocopherols were quantified following extraction in Vaseline oil, which was used to avoid the effects of tocopherols present in plant oils [53,54]. Leaf samples were extracted, clarified via centrifugation, and absorbance was measured at 292 nm using α-tocopherol as a reference standard. Tocopherol content was calculated as total vitamin E equivalents and expressed relative to sample mass.

### 2.9. Statistical Analysis

Statistical analysis of the results was performed using the GraphPad Prism 8.0.1 software package by ANOVA with Tukey’s multiple comparison test. Data are presented as mean ± standard deviation (SD) based on at least three biological replicates (n ≥ 3).

## 3. Results

### 3.1. Leaves’ Oxidative Status and DNA Damage After Temperature Pre-Exposure and TBSV Infection

**Figure 1:**
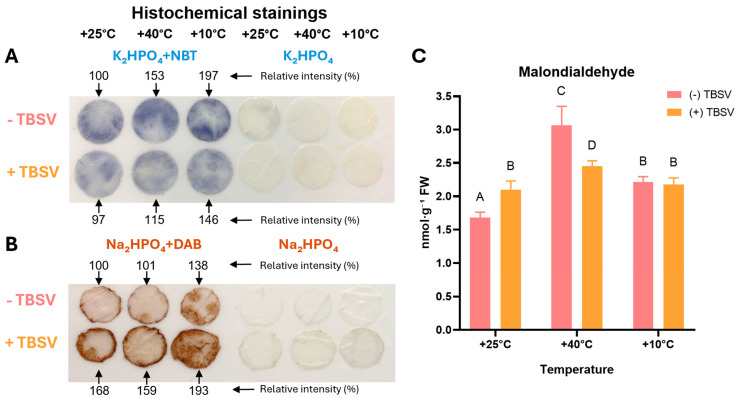
The influence of temperature pre-exposure and TBSV infection on plants’ oxidative status in *N. benthamiana* leaves. Histochemical staining using NBT (**A**) to visualize O_2_^•−^ (blue-violet staining) and DAB (**B**) to visualize H_2_O_2_ (brown staining) with relative intensity (%) measured using ImageJ. Relative activities are expressed as percentage of the +25 °C mock-inoculated control (**C**) Malondialdehyde (MDA) concentration, nmol·g^−1^ FW (n = 5, mean ± SD). Different capital letters indicate statistically significant differences between groups at *p* < 0.05 (ANOVA with Tukey’s multiple comparison test). (-) TBSV—mock-inoculated; (+) TBSV—TBSV infected.

**Figure 2:**
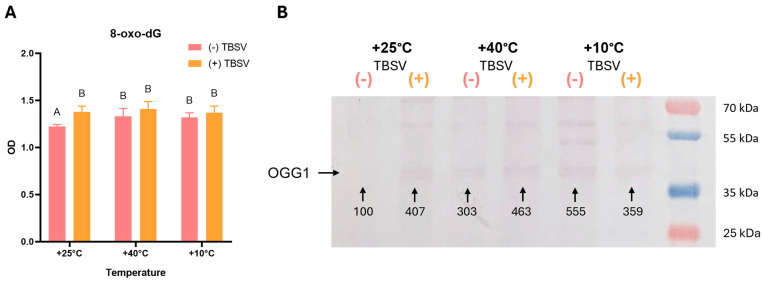
Analysis of oxidative DNA damage (8-oxo-dG) and OGG1 enzyme expression after temperature stress and TBSV infection in *N. benthamiana* leaves. (**A**) 8-oxo-dG level, OD (n = 5, mean ± SD). Different capital letters indicate statistically significant differences between groups at *p* < 0.05 (ANOVA with Tukey’s multiple comparison test). (**B**) Western blot analysis to visualize OGG1 protein abundance and relative intensity (%) competed to control (numbers below the bands). This figure displays one representative experiment from two independent Western blot analyses. (-) TBSV—mock-inoculated; (+) TBSV—TBSV infected. Western blot original images can be found in Supplementary Materials.

In the first stage, the *N. benthamiana* leaves’ oxidative status was analyzed after pre-exposure to temperatures and subsequent TBSV infection (Figure 1 and Figure 2). Oxidative status was assessed based on O_2_^•−^ and H_2_O_2_ accumulation, as well as LPO intensity determined by MDA content (Figure 1). For evaluating oxidative DNA damage, 8-oxo-dG levels and OGG1 activity were analyzed (Figure 2).

O_2_^•−^ levels (Figure 1A) in uninfected plants showed a pronounced increase in NBT signal intensity following temperature treatments compared to the control. The highest accumulation was observed in plants pre-exposed to 10 °C (~197% of control), while after 40 °C, it was ~153%. Interestingly, O_2_^•−^ accumulation was reduced in TBSV-infected plants compared to uninfected ones after all temperature treatments. However, in plants previously exposed to 10 °C and 40 °C, the NBT signal remained elevated relative to the control, indicating a persistent effect of prior temperature stress.

Unlike O_2_^•−^, H_2_O_2_ accumulation demonstrated the opposite trend (Figure 1B). TBSV infection consistently increased DAB signal intensity relative to mock-inoculated plants. H_2_O_2_ levels in infected plants at 25 °C were approximately 68% higher than in the uninfected control. Notably, pre-exposure to 40 °C alone did not significantly affect H_2_O_2_ accumulation, with levels remaining close to control (~101%), and infected plants showing comparable levels at 25 °C and 40 °C (~159%). In contrast, cold pre-exposure increased H_2_O_2_ levels to ~138%, and subsequent TBSV infection resulted in the highest accumulation (~193%). TBSV infection induced a sustained increase in H_2_O_2_ levels regardless of the previous temperature treatments, with the most pronounced accumulation occurring in *N. benthamiana* pre-exposed to cold.

MDA levels showed a significant residual effect on LPO following prior temperature stress (Figure 1C). In mock-inoculated plants, pre-exposure to 40 °C resulted in the highest residual MDA levels, reaching nearly twice the 25 °C control levels. Interestingly, subsequent TBSV infection attenuated this effect, leading to a statistically significant decrease in MDA levels (*p* < 0.05). In contrast, TBSV at 25 °C infection increases MDA levels to ~30% compared to the control (*p* < 0.05). However, in the cold-exposed group at 10 °C, MDA content remained similar between infected and mock-inoculated plants, and these levels were comparable to those observed in TBSV-infected plants at 25 °C (*p* > 0.05).

Oxidative DNA damage analysis revealed that 8-oxo-dG levels increased both after temperature stress and TBSV infection in *N. benthamiana* (Figure 2A). Despite a general trend toward higher 8-oxo-dG accumulation in infected plants compared to mock-inoculated samples, these differences were not statistically significant (*p* > 0.05). However, OGG1 protein levels showed more pronounced dynamics (Figure 2B). Pre-exposure to heat (40 °C) and cold (10 °C) significantly induced OGG1 accumulation in mock-inoculated plants, with the highest protein level observed after cold exposure (~555%). Subsequent TBSV infection further stimulated an increase in OGG1 levels in the control and 40 °C groups, while a decrease was observed in the 10 °C treatment.

### 3.2. Protein and Enzymatic Stress Responses in Leaves After Temperature Pretreatment and TBSV Infection

**Figure 3:**
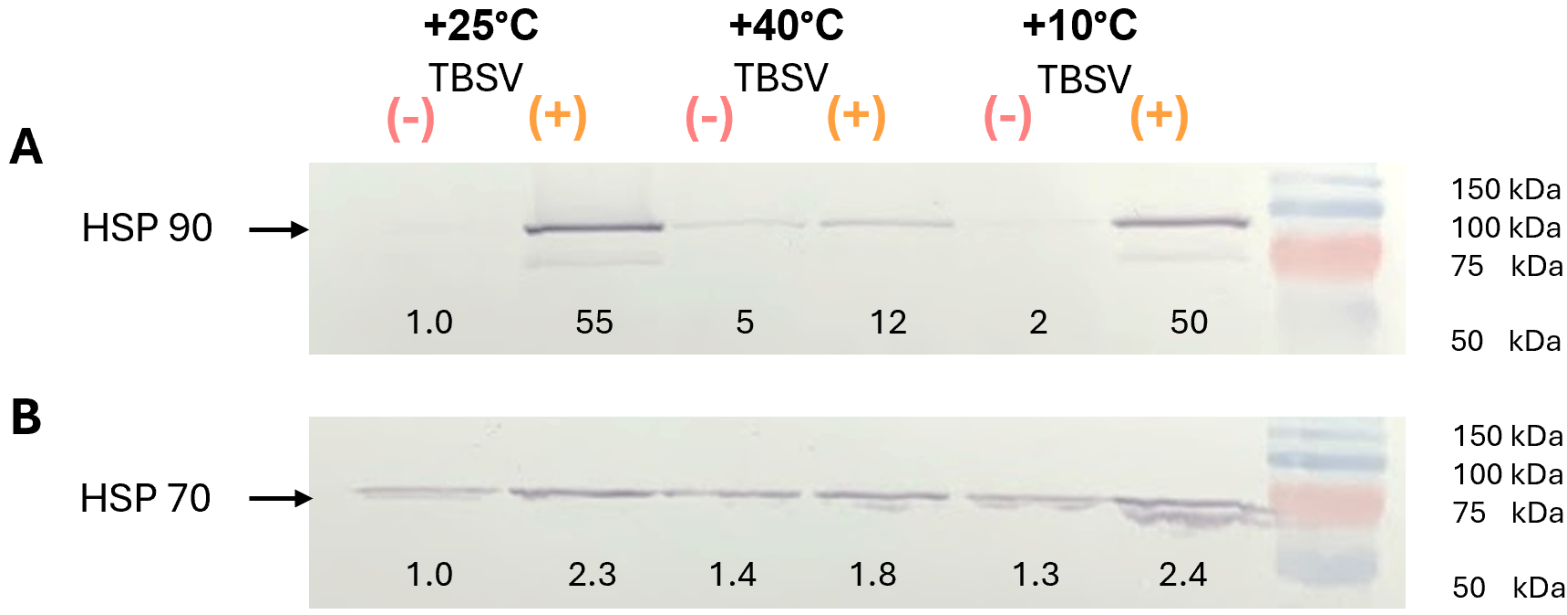
Expression of heat shock proteins (HSP90 and HSP70) in *N. benthamiana* leaves after temperature stress and TBSV infection. Western blot analysis for visualization of HSP90 (**A**) and HSP70 (**B**) protein expression. Numbers below the bands correspond to relative intensities calculated based on densitometry. These figures display one representative experiment from two independent Western blot analyses. (-) TBSV—mock-inoculated; (+) TBSV—TBSV infected. Western blot original images can be found in Supplementary Materials.

**Figure 4:**
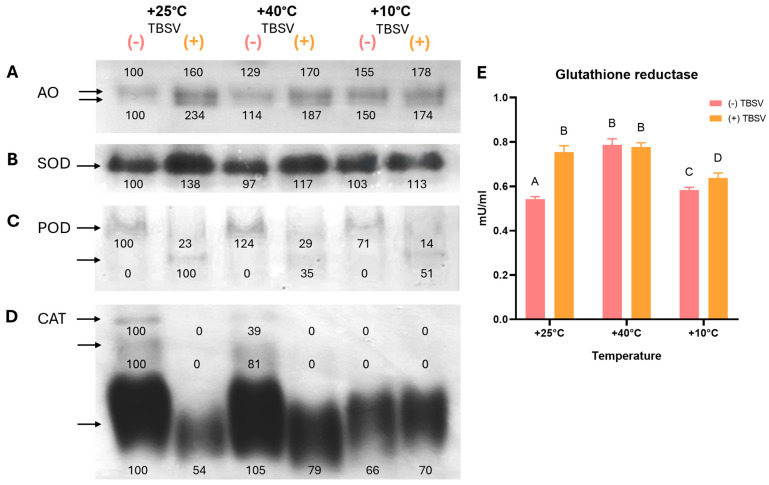
The influence of temperature pre-exposure and TBSV infection on redox-dependent enzymes in *N. benthamiana* leaves. (**A**–**D**) Native-PAGE in-gel activity analysis of aldehyde oxidase (AO), superoxide dismutase (SOD), peroxidase (POD), and catalase (CAT) activities. Numbers above or below the bands indicate relative activity intensities expressed as a percentage of the 25 °C mock-inoculated control. For the infection-specific lower POD activity band, relative intensity was calculated with respect to the 25 °C TBSV-infected sample, as this band was not detectable in mock-inoculated plants. Each panel shows one representative gel from at least two independent native-PAGE activity assays. (**E**) Glutathione reductase (GR) activity was determined spectrophotometrically (n = 3, mean ± SD). Different capital letters indicate statistically significant differences between groups at *p* < 0.05 (ANOVA with Tukey’s multiple comparison test). (-) TBSV—mock-inoculated; (+) TBSV—TBSV infected. Native-PAGE in-gel activity original images can be found in Supplementary Materials.

The observed changes in ROS levels, LPO intensity, oxidative DNA damage, and DNA repair responses indicated profound alterations in cellular homeostasis. To further characterize these stress-associated responses, key protein and enzymatic components were analyzed. Particular attention was given to HSP70 and HSP90 as important components for maintaining protein stability and stress signaling (Figure 3). The activities of AO and antioxidant enzymes SOD, POD, CAT, and GR were examined (Figure 4) to evaluate enzymatic redox regulation after temperature pre-exposure and TBSV infection.

Analysis of HSP90 and HSP70 levels revealed different accumulation dynamics in response to stress factors (Figure 3). HSP90 levels demonstrated a significant dependence on both temperature pretreatment and TBSV infection (Figure 3A). Heat stress alone resulted in a 5-fold induction of HSP90 in mock-inoculated plants, while cold stress resulted in a 2-fold increase. Subsequent TBSV infection resulted in a significant accumulation of this chaperone, with levels increasing 50- and 55-fold in the 10 °C and 25 °C groups, respectively. However, heat pre-exposure apparently significantly limited the HSP90 response to subsequent infection, as evidenced by a 12-fold increase, which is lower than in the 10 °C and 25 °C groups.

HSP70 dynamics were characterized by more moderate changes in all studied variants (Figure 3B). Preliminary temperature treatment resulted in a slight increase in protein levels in uninfected plants by 1.4 times at 40 °C and 1.3 times at 10 °C. Subsequent infection with TBSV stimulated HSP70 accumulation in all temperature regimes. Moreover, the maximum protein level was observed in the 10 °C (+TBSV) and 25 °C (+TBSV) variants, where it increased by 2.4 and 2.3 times, respectively, compared to the control. In the 40 °C (+TBSV) group, HSP70 content increased by 1.8 times, which was slightly lower than the values in the other infected groups.

Native-PAGE in-gel activity analysis revealed two electrophoretically distinct AO-related activity bands in *N. benthamiana* leaves (Figure 4A). In uninfected plants, any deviation from the optimal temperature led to an increase in AO activity. Specifically, the most significant induction occurred after cold exposure, where activity reached approximately 155% and 150% for the upper and lower activity bands, respectively. Preliminary exposure to heat also stimulated AO activity, though to a lesser extent (129% and 114%). Subsequent TBSV infection triggered a further increase in AO activity, though the intensity of this response depended on prior temperature pre-exposure. Under control conditions, infection induced a pronounced increase in the lower AO-related activity band, reaching approximately 234% of the control. In contrast, in plants previously exposed to 40 °C or 10 °C AO activity in infected samples increased to a lesser extent (~170–187%) relative to the control.

SOD activity demonstrated a predominant association with TBSV infection. In mock-inoculated *N. benthamiana*, SOD activity remained relatively stable after temperature pre-exposure and was not significantly different from the control. In contrast, subsequent TBSV infection was accompanied by a sustained increase in SOD activity across all temperature treatments. The highest induction was recorded in plants previously maintained at the optimal temperature with TBSV (~138%). Temperature pre-exposure notably attenuated the SOD response to infection, with activity levels rising to 117% (40 °C) and 113% (10 °C) compared to the control.

In-gel analysis of POD activity revealed two electrophoretically distinct bands, whose activities were specifically altered by temperature pre-exposure and TBSV infection (Figure 4C). In uninfected plants, the upper POD activity band predominated, with its activity increased to 124% after heat exposure and decreased to 71% after cold exposure relative to the control. Following this, TBSV infection triggered a pronounced restructuring of the POD profile. The upper band was rapidly suppressed across all temperature regimes (14–29% of the control), while an infection-induced lower band appeared, which was absent in healthy plants. The highest activity of this virus-induced band was recorded at 25 °C (100%), whereas prior heat and cold treatments significantly reduced its activity to 35% and 51%, respectively.

CAT activity revealed a complex profile consisting of three electrophoretically distinct bands, the activity of which was profoundly repressed upon infection (Figure 4D). In mock-inoculated plants at 25 °C, the full complement of CAT activity bands constituting the total enzymatic pool was detected, whereas prior temperature stress alone reduced overall CAT activity. Heat exposure resulted in partial loss of the upper bands (39% and 81% of control) while maintaining activity of the lower band (105%), while cold caused the strongest suppression of the CAT enzymatic system. TBSV infection induced a strong repression of CAT activity, regardless of temperature pretreatment. The upper and intermediate CAT activity bands were completely suppressed (0%) in all infected samples. Only the most stable lower CAT activity band retained measurable activity, and its intensity depended on temperature pre-exposure. Following infection at 25 °C, its activity decreased to approximately 54% of the control, whereas prior exposure to 40 °C allowed partial preservation of this activity (approximately 79%). In contrast, after cold pre-exposure, the activity of this band was minimal. Therefore, viral infection resulted in near-complete inhibition of the CAT defense system, while preliminary heat hardening permitted partial maintenance of the functionality of a single CAT activity band.

GR activity responded differentially to temperature pre-exposure and TBSV infection (Figure 4E). In mock-inoculated plants, the strongest increase in GR activity was observed after 40 °C pre-exposure (0.79 mU/mL), reaching levels comparable to those measured in TBSV-infected plants at 25 °C (0.76 mU/mL), where the control level was 0.54 mU/mL. In contrast, cold pre-exposure did not have a substantial effect on basal GR activity. The effect of TBSV infection on GR activity depended on prior temperature treatment. At 25 °C and after 10 °C, TBSV infection resulted in an increase in GR activity. In contrast, in plants pre-exposed to 40 °C after infection GR activity remained at a level similar (0.78 mU/mL) to that induced by heat pretreatment alone, indicating a limited additional effect of TBSV under these conditions.

### 3.3. Changes in Secondary Metabolites and Non-Enzymatic Antioxidants After Preliminary Temperature Stress and TBSV Infection

**Figure 5:**
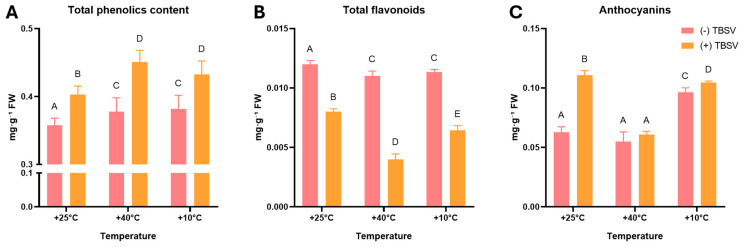
Phenolic compounds content changes under TBSV infection and after temperature stress. (**A**) Total phenolic, (**B**) total flavonoids, and (**C**) anthocyanin content, mg·g^−1^ FW. Data are presented as mean ± SD based on five biological replicates (n = 5). Different capital letters indicate statistically significant differences between groups at *p* < 0.05 (ANOVA with Tukey’s multiple comparison test). (-) TBSV—mock-inoculated; (+) TBSV—TBSV infected.

**Figure 6:**
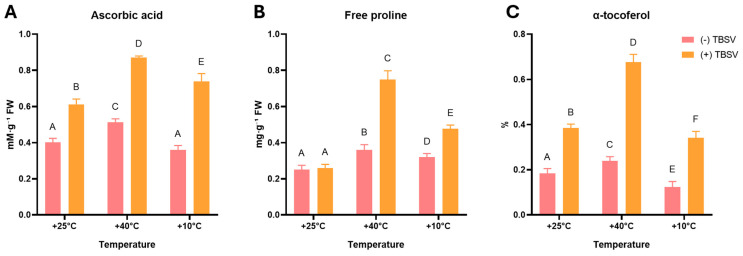
Analysis of non-enzymatic antioxidant content of *N. benthamiana* leaves under TBSV infection and after temperature stress. (**A**) Ascorbic acid, mM·g^−1^ FW, (**B**) free proline, mg·g^−1^ FW, and (**C**) α-tocopherol. Data are presented as mean ± SD based on five biological replicates (n = 5). Different capital letters indicate statistically significant differences between groups at *p* < 0.05 (one-way ANOVA, Tukey’s multiple comparison test). (-) TBSV—mock-inoculated; (+) TBSV—TBSV infected.

Given the observed restructuring of the enzymatic component, the next step was to evaluate the non-enzymatic components of the defense. To characterize the redox status and the metabolic response of secondary metabolism, a pool of phenolic compounds, including TPC, flavonoids, and anthocyanins, was analyzed (Figure 5). To assess the capacity of the ROS detoxification system, the contents of AsA, free proline, and α-tocopherol were assessed (Figure 6).

Secondary metabolic analysis revealed significant changes in the phenolic profile of *N. benthamiana* leaves in response to temperature pretreatment and TBSV infection (Figure 5). TPC increased during infection in all experimental groups, while thermal pretreatment alone did not induce stronger accumulation than infection (Figure 5A). Under control conditions, infection resulted in an increase in TPC from 0.36 to 0.40 mg·g^−1^ FW. Maximum values were observed under heat pretreatment combined with TBSV infection, while cold pretreatment with infection also resulted in significantly elevated TPC (*p* < 0.05).

Unlike TPC, flavonoid content showed a pronounced decrease following TBSV infection (Figure 5B). In uninfected plants, flavonoid levels remained relatively stable across all temperature treatments (0.011–0.012 mg·g^−1^ FW). However, infection resulted in a significant decrease in their content, most pronounced after heat pre-exposure, where flavonoid levels dropped to 0.004 mg·g^−1^ FW (*p* < 0.05). After pre-exposure at 10 °C, the flavonoid content in TBSV-infected plants also significantly decreased to 0.0065 mg·g^−1^ FW (*p* < 0.05), remaining higher than in the infected group after 40 °C.

Anthocyanins, in contrast to TPC and flavonoids, exhibited a stronger temperature-dependent regulation of the response to TBSV infection (Figure 5C). Cold exposure alone was associated with increased anthocyanin levels compared to controls, while subsequent TBSV infection only slightly increased anthocyanin content (*p* < 0.05). Under TBSV infection conditions at 25 °C, anthocyanin content increased from 0.063 to 0.11 mg·g^−1^ FW. In contrast, pre-exposure to heat and subsequent infection with TBSV did not result in an increase in anthocyanin levels compared to the mock-inoculated *N. benthamiana* leaves (*p* > 0.05).

The AsA content in uninfected plants increased moderately after heat pre-exposure and remained virtually unchanged after cold exposure compared to the control (Figure 6A). TBSV infection was accompanied by a marked increase in AsA levels in all experimental treatments. At the optimal temperature, the AsA concentration increased from 0.40 to 0.61 mM·g^−1^ FW. Heat pretreatment enhanced this response, leading to an increase in ascorbate content to 0.87 mM·g^−1^ FW. At the same time, pretreatment at 10 °C limited the response to 0.74 mM·g^−1^ FW, which was nevertheless significantly higher than in mock-inoculated plants.

Free proline dynamics showed the most contrasting changes (Figure 6B). In the absence of infection, pre-exposure to 40 °C and 10 °C caused a statistically significant but moderate accumulation of proline (0.32–0.36 mg·g^−1^ FW). No additional increase in proline content was observed after infection at 25 °C (*p* > 0.05). In contrast, in the heat-primed group, infection resulted in a strong increase in proline content to 0.75 mg·g^−1^ FW, which was approximately three times higher than the level in the uninfected control *N. benthamiana*. Cold pretreatment also promoted an increase in proline during infection to 0.47 mg·g^−1^ FW, but this level remained significantly lower than the values recorded after heat pre-exposure.

In uninfected plants, the α-tocopherol content varied depending on the temperature pre-exposure and ranged from 0.12 to 0.24% (Figure 6C). Heat pretreatment was associated with an increase in α-tocopherol levels, whereas cold resulted in their reduction. TBSV infection led to an increase in α-tocopherol content after all temperature conditions in *N. benthamiana* (*p* < 0.05). The most pronounced response was observed in the 40 °C (+TBSV) group, where α-tocopherol levels reached 0.68%. In the +10 °C (+TBSV) variant, α-tocopherol content (0.34%) was lower than in infected plants maintained at 25 °C (0.38%) and was markedly lower than the level recorded after heat pre-exposure.

## 4. Discussion

This study was designed to determine how temperature pretreatment alters the redox and defense responses of *N. benthamiana* during subsequent infection with TBSV. Temperature pretreatment does not simply enhance or attenuate stress responses but rather fundamentally alters the redox, metabolic, and proteostatic context within which viral infection develops. The results indicate that the outcome of TBSV infection depends not only on the virus itself but also on the physiological state of the host plant before inoculation.

### 4.1. Temperature-Dependent Reorganization of the Redox Phenotype

TBSV infection in *N. benthamiana* induces alterations in cellular redox status that are strongly influenced by temperature pretreatment. Analysis of ROS accumulation revealed distinct responses of O_2_^•−^ and H_2_O_2_ during infection. O_2_^•−^ levels remained elevated after both cold and heat pretreatment compared to the control but were consistently reduced in TBSV-infected plants relative to the corresponding mock-infected plants. In contrast, H_2_O_2_ accumulation increased in response to TBSV infection across under all temperature conditions, with comparable H_2_O_2_ levels at 25 °C and 40 °C and the highest accumulation after cold pretreatment. These differences suggest that temperature pretreatment creates a different redox state in which TBSV infection progressing under different oxidative conditions rather than through a single stress pathway [55,56]. Thus, temperature and viral infection differentially affect individual ROS rather than uniformly enhancing oxidative stress [55,56].

### 4.2. Changes in HSP70 and HSP90 Proteins During Viral Integration After Temperature Exposure

In addition to temperature-dependent reorganization of oxidative balance during TBSV infection, pronounced changes in the host chaperone system were observed [57,58,59]. Heat pretreatment alone induced HSP90 accumulation in mock-inoculated plants, whereas pretreatment at 10 °C caused a moderate increase. Under optimal temperature conditions, TBSV infection triggered a strong HSP90accumulation accompanied by a moderate induction of HSP70, indicating activation of the host protein protective mechanism. A comparable HSP profile was observed in cold-pretreated plants, where infection-induced HSP90 and HSP70 levels were similar to those detected at 25 °C. In contrast, despite elevated basal HSP90 levels following heat pretreatment, subsequent TBSV infection resulted in a weaker additional induction of both chaperones, particularly HSP90. Functionally, HSP90 is more often associated with the stabilization and activation of regulatory target proteins and replication-related complexes, whereas HSP70 is more often associated with protein folding, refolding, and prevention of aggregation [60,61,62,63]. Therefore, the weaker virus-associated induction after heat pretreatment may indicate not only a reduced general chaperone response [60,61] but also a limitation in the formation or maintenance of protein complexes required for efficient viral infection and signaling [61,62,63].

### 4.3. Metabolic Plasticity and Non-Enzymatic Antioxidant Responses

TBSV infection after different temperature pre-exposures was associated with changes in secondary metabolites and non-enzymatic antioxidants. TBSV infection caused a sustained increase in TPC after temperature pretreatments, indicating activation of phenylpropanoid pathways as a common component of the antiviral response [64,65,66]. In contrast, the decrease in flavonoids suggests a selective redirection of metabolic resources, especially after heat treatment [3]. Anthocyanin accumulation further highlights the contrast between cold and heat responses. While cold stress promoted their synthesis [65], heat pretreatment suppressed anthocyanin overaccumulation during TBSV infection [67,68]. These results suggest that temperature pretreatment defines the metabolic context of TBSV infection by permitting or restricting specific branches of secondary metabolism.

In parallel, non-enzymatic antioxidants with dual signaling and redox roles also showed temperature-dependent accumulation in TBSV-infected plants [69,70,71,72]. AsA content increased in all infected plants, with the strongest rise after heat pretreatment. Free proline showed an even stronger dependence on temperature pre-exposure, with no change during infection at the optimal temperature but increasing following 40 °C and 10 °C. Together, these coordinated changes indicate that temperature pretreatment alters metabolic pathways during viral infection, promoting the accumulation of redox-active metabolites involved in ROS scavenging and stress-related signaling [69,70,71,72]. These coordinated responses also suggest that heat pretreatment creates conditions that alter redox metabolism, leading to changes in antioxidants and signaling conditions during subsequent TBSV infection.

### 4.4. Mechanisms of Macromolecular Protection and Aldehyde Detoxification

However, despite temperature-dependent changes in redox-mediated metabolites and non-enzymatic antioxidants, oxidative stress was not completely neutralized during TBSV infection. Oxidative DNA damage was observed under combined temperature and viral stress, as reflected by increased 8-oxo-dG accumulation in mock-infected plants after temperature exposure as well as upon TBSV infection [73,74]. These changes were accompanied by altered OGG1 levels, consistent with activation of base excision repair mechanisms [75,76].

Similar protective responses were observed at the membrane integrity. Temperature pre-conditioning increased α-tocopherol content to membrane protection [77,78], while TBSV infection at 25 °C and 40 °C induced less accumulation. This may contribute to the lower LPO level in infected plants after heat pretreatment compared with the strong increase caused by 40 °C alone [77,78]. After cold stress, subsequent TBSV infection did not further alter LPO, resulting in MDA levels comparable to those in infected plants at the optimal temperature. This stabilization of LPO coincides with the GR activity [79,80], which remains responsive after cold pre-exposure but does not exceed the levels observed at 25 °C during infection. In contrast, the reduction in MDA accumulation after TBSV infection at 40 °C appears to be more closely associated with α-tocopherol than with GR. This suggests that temperature pre-conditioning alters the way viral infection is integrated into the pre-existing redox and protective mechanisms.

Within these temperature-dependent redox mechanisms, AO may act as a key link between ROS generation and lipid-derived aldehyde metabolism [81]. We hypothesize that AO induction during TBSV infection enhances the removal of toxic aldehyde pools, consistent with the reduced MDA levels observed during infection after heat pretreatment compared to heat stress alone [35,43,81]. This also suggests that high MDA levels may limit efficient viral dissemination, making aldehyde accumulation a potential restrictive factor for TBSV infection [82,83,84,85]. At 25 °C, strong oxidative stress TBSV infection coincided with efficient AO activation and limited MDA accumulation, suggesting that aldehyde detoxification helps maintain conditions permissive for infection. After cold pretreatment, AO activity was also high, whereas MDA remained at levels comparable to those in infected plants at 25 °C. Thus, these observations support the idea that AO activity stabilizes aldehyde levels within a tolerable range rather than stimulating LPO during TBSV infection.

### 4.5. Enzymatic Control of the Permissive Redox Phenotype

In addition to aldehyde metabolism, AO activity generates ROS as a secondary by-product, requiring precise control of O_2_^•−^ and H_2_O_2_ levels during TBSV infection [10,42,46]. Infection was associated with decreased O_2_^•−^ accumulation, closely correlated with changes in SOD activity [86], indicating tight regulation of O_2_^•−^ levels during infection. In contrast, H_2_O_2_ appears to obey a different regulatory logic during TBSV infection. H_2_O_2_ accumulation increased after infection, suggesting a signaling-related role rather than just a consequence of oxidative stress. This is supported by the induction of infection-associated POD activity, detected only after virus inoculation, although the virus-specific band decreased during infection following heat pretreatment [87,88,89]. Changes in CAT activity further support active regulation of the cellular H_2_O_2_ pool. Viral infection caused a strong decrease in CAT activity, accompanied by a loss of two catalase isoforms, which was also observed after cold pretreatment and subsequent infection [90,91,92,93]. In contrast, heat pretreatment only partially attenuated CAT activity and maintained the constitutive catalase isoform at a higher level than during infection at 25 °C. Overall, differences in CAT and POD activities indicate that H_2_O_2_ is not consistently neutralized during TBSV infection but rather is regulated for signaling functions.

### 4.6. Temperature Pretreatment as a Modifier of Viral Pathogenesis

These results indicate that TBSV infection does not simply induce oxidative stress but is associated with reorganization of the host plant redox state, signaling, and metabolic pathways, thereby shaping a permissive redox phenotype. In this model, prior temperature exposure in *N. benthamiana* acts as a key modifier by setting the initial redox and proteostatic state in which viral infection develops. Rather than causing a uniform increase in oxidative damage, TBSV infection is associated with regulation of individual ROS, differential HSP activation, redistribution of secondary metabolism, and control of antioxidant and reparative mechanisms. These changes indicate that the TBSV utilizes host redox signaling pathways and the metabolic plasticity of the host to optimize the conditions for its replication, while the prior temperature experience determines how effectively this reconfiguration can proceed. Thus, temperature pretreatment does not simply increase or decrease viral stress but qualitatively changes how infection is integrated into the plant’s existing protective and regulatory mechanisms.

## 5. Conclusions

This study examined how preliminary temperature exposure influences the interaction between plants and viruses. We found that both heat and cold pretreatments significantly alter the redox states of *N. benthamiana*, which affects the nature and outcome of subsequent TBSV infection. Our results suggest that the accumulation of LPO and O_2_^•−^ acts as a limiting factor for viral development. We observed that TBSV infection was associated with reduced levels of O_2_^•−^ under all temperature conditions and a decrease in MDA content after heat pretreatment. The decrease in MDA after heat stress coincided with increased AO activity, indicating enhanced detoxification of toxic lipid-derived aldehydes. Additionally, the activity of GR and the accumulation of α-tocopherol further support the role of membrane-protective antioxidant systems. These changes in oxidation and reduction processes correspond to responses driven by signaling rather than those caused by cellular damage. TBSV alters redox signaling and enzymatic regulation to create permissive conditions within cells, leading to a controlled ROS profile that is characterized by low O_2_^•−^ levels and high H_2_O_2_ levels. Moreover, heat pretreatment may limit viral development compared to optimal temperatures or cold pre-exposure, likely through modification of the host redox and proteostatic state, including reduced infection-associated HSP accumulation after heat stress. These findings reveal that the outcome of TBSV infection depends on the host cell physiological state established before inoculation, particularly on redox homeostasis and associated protective mechanisms, which may either limit the effectiveness of viral development or be modulated by the virus to support replication.

## Data Availability

The original contributions presented in this study are included in the article/Supplementary Materials. Further inquiries can be directed to the corresponding authors.

## Supplementary Materials

The following supporting information can be downloaded at: https://www.mdpi.com/article/10.3390/biom16030446/s1. The original Western blot and Native-PAGE in-gel activity images can be found in the Supplementary Materials.
