# Supplementary figures and images for: TBSV Alters Host Redox State After Short-Term Temperature Pre-Exposure in Nicotiana benthamiana
Source: Biomolecules. 2026 Mar 17;16(3):446. doi: 10.3390/biom16030446 (PMC13024633; doi:10.3390/biom16030446)

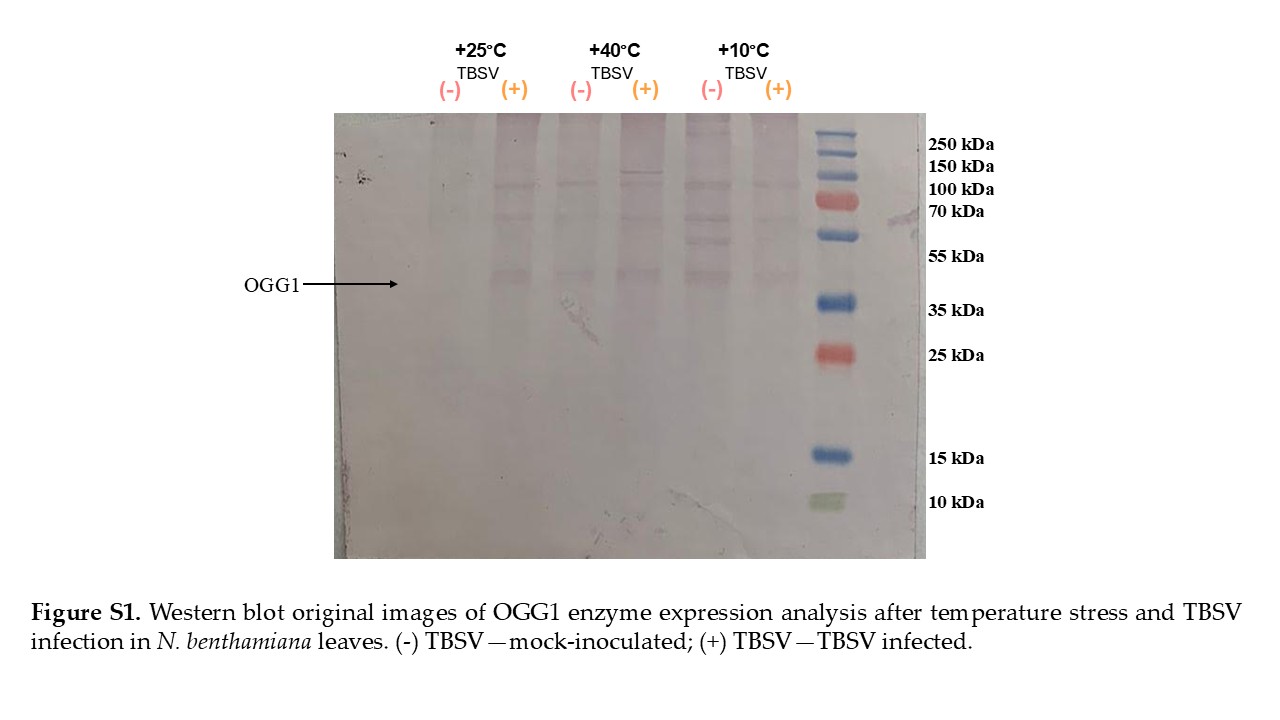

Supplement: Supplementary file 1 [file biomolecules-16-00446-s001.zip › Figure S1.JPG]

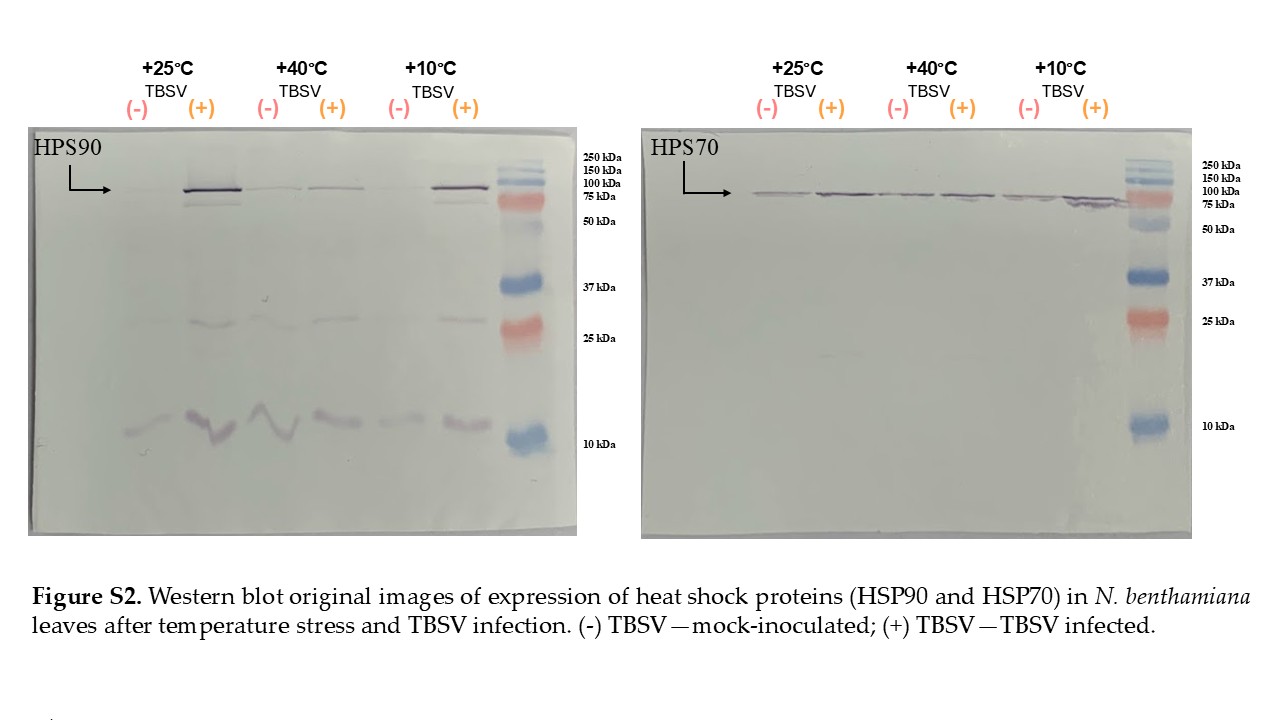

Supplement: Supplementary file 1 [file biomolecules-16-00446-s001.zip › Figure S2.JPG]

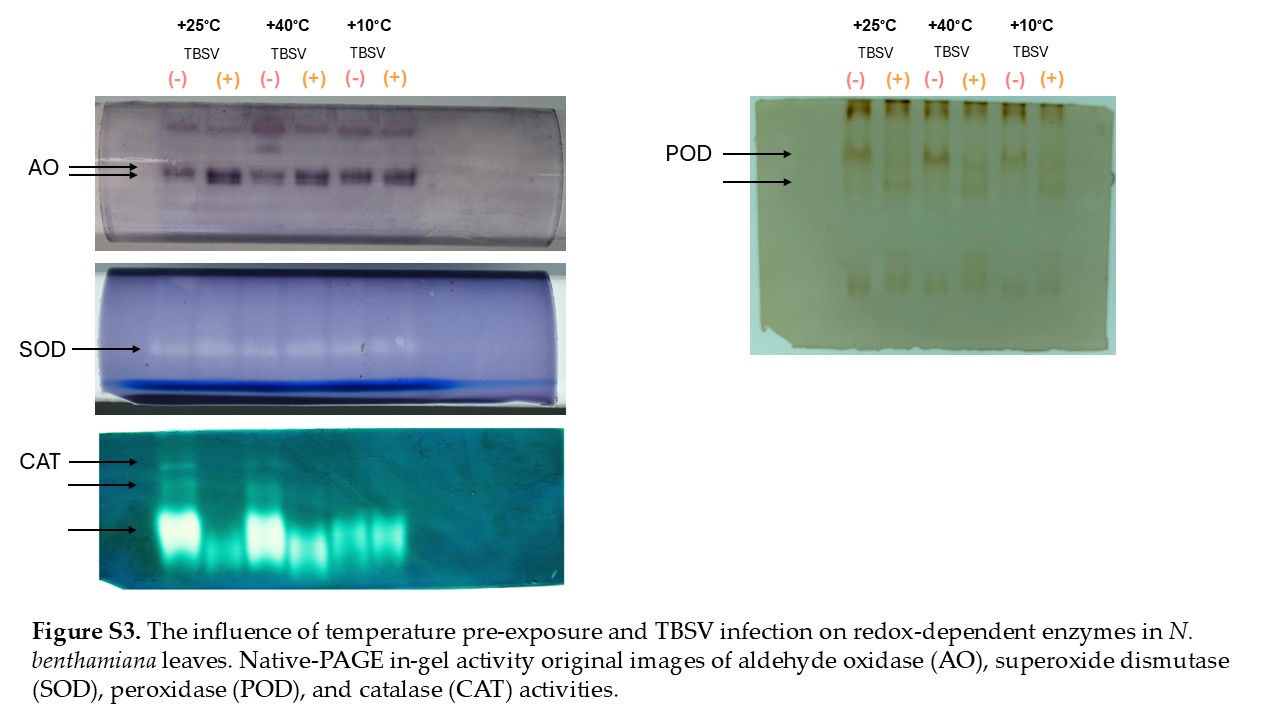

Supplement: Supplementary file 1 [file biomolecules-16-00446-s001.zip › Figure S3.JPG]
